# Estradiol levels are elevated in older men with diffuse cutaneous SSc and are associated with decreased survival

**DOI:** 10.1186/s13075-019-1870-6

**Published:** 2019-04-02

**Authors:** DeAnna Baker Frost, Bethany Wolf, Christine Peoples, Jessica Fike, Katherine Silver, Maureen Laffoon, Thomas A. Medsger, Carol Feghali-Bostwick

**Affiliations:** 1 Department of Medicine, Division of Rheumatology and Immunology, Medical University of South Carolina, 96 Jonathan Lucas Street, Charleston, SC 29425 USA; 2 College of Medicine, Department of Public Health Sciences, Medical University of South Carolina, 96 Jonathan Lucas Street, Charleston, SC 29425 USA; 3 Department of Medicine, Division of Rheumatology and Clinical Immunology, University of Pittsburgh, 3500 Terrace Street, Pittsburgh, PA 15261 USA

**Keywords:** Systemic sclerosis, Estrogen, Fibrosis, Survival

## Abstract

**Background:**

Systemic sclerosis (SSc) is a female-predominant disease, characterized by excessive extracellular matrix deposition (ECM) with dermal and internal organ fibrosis. Considering the sex-based disparity in disease incidence, estradiol (E2), an estrogen form with pro-fibrotic effects, may play a role in SSc. We reported that post-menopausal women with diffuse cutaneous (dc)SSc have higher serum E2 levels compared to similar aged, healthy controls. Since males with SSc tend to have more severe disease, we examined serum E2 in dcSSc males in relation to disease characteristics and survival.

**Methods:**

We measured serum E2 in 83 dcSSc men > 50 years old from the University of Pittsburgh Scleroderma Center and similar aged healthy controls. Using statistical modeling, we examined the associations between serum E2, internal organ involvement, autoantibody profiles, and survival.

**Results:**

Male dcSSc patients had significantly higher serum E2 levels compared to healthy males and similar aged dcSSc post-menopausal women. Male dcSSc patients with high serum E2 had significantly more heart involvement, a trend for higher skin thickness progression rate, and worse survival. Using Cox regression modeling, increased serum E2 levels in anti-Scl-70 antibody-positive dcSSc males were associated with an increased risk of death.

**Conclusions:**

dcSSc males > 50 years old have higher levels of serum E2 compared to healthy controls and dcSSc post-menopausal women. Elevated serum E2 levels in dcSSc males are associated with heart involvement, trend to progression of dermal fibrosis, and, if anti-Scl-70 antibody positive, worse survival. Our study expands on previous work implicating E2 in dermal fibrosis in SSc and associates E2 levels with internal organ involvement and survival. These data suggest a role for estrogen imbalance in dcSSc.

## Background

Systemic sclerosis (SSc) is an autoimmune, connective tissue disease of unknown etiology characterized by immune system dysregulation and excessive extracellular matrix (ECM) synthesis [1, 2]. Clinical classification of SSc includes limited cutaneous (lc)SSc and diffuse cutaneous (dc)SSc forms [1]. Both variants manifest with cutaneous fibrosis, but dcSSc results in significant morbidity and mortality [3–5]. One study has shown that an increased skin score is related to increased pain and quality of life impairment [6]. Typically, patients with dcSSc have higher skin scores, due to more extensive skin involvement, compared to patients with lcSSc. Currently, there is no clinically available therapy for effectively reducing excessive ECM production in SSc.

As in most autoimmune diseases, there is a female predominance in SSc with a female to male ratio of 3:1, which increases to 9:1 during childbearing years [1]. However, male patients have more severe disease compared to women [7]. Men have more severe interstitial lung disease (ILD), which is currently the leading cause of death in SSc patients [8] and significantly reduces survival [7, 8].

Differences noted in disease incidence and severity between sexes suggest that hormonal influence may affect the development, progression, and severity of SSc. One major hormone likely contributing to these differences is estradiol (E2). E2, one of the forms of estrogen, is found in the serum of non-pregnant females, with higher levels during the childbearing years and reduced levels after menopause [9]. Thus, circulating E2 levels are highest during the time when the SSc F to M incidence ratio is higher [1], suggesting a possible link between SSc development and E2 levels. Healthy, older males have higher levels of circulating E2 than post-menopausal females, predominantly due to the conversion of E2 from testosterone. The conversion of testosterone to E2 occurs through aromatization by the enzyme aromatase. With aging, older males also have increased fat mass, and since aromatase is expressed in fat, there is greater E2 conversion [10].

In a previous study, we reported that post-menopausal female dcSSc patients have significantly higher levels of serum estrogens compared to age-matched healthy volunteers [11]. A subsequent study measuring serum E2 and testosterone levels in post-menopausal SSc patients confirmed higher serum E2 levels in post-menopausal dcSSc patients but significantly lower serum testosterone levels compared to healthy volunteers [12]. However, serum E2 levels in dcSSc males of comparable age to post-menopausal women have not been evaluated. We therefore measured E2 levels in serum samples from dcSSc males > 50 years old collected at the University of Pittsburgh Scleroderma Center. We hypothesized that serum E2 levels would be elevated in male dcSSc patients > 50 years old and would be associated with clinical features of the disease.

## Methods

### Patient selection

Patients were recruited at the University of Pittsburgh Scleroderma Center. We used the following criteria for subject selection: All male patients had definite dcSSc at the time of the first visit to the University of Pittsburgh from 1978 to 2009 or developed diffuse skin changes within the 6 months after the first visit and had a first visit serum sample available for analysis. Male dcSSc patients were age > 50 years old at the time of the first visit and had disease duration from first SSc symptom of 2.0 years or less. Healthy male controls age 50 or older were collected at the University of Pittsburgh over the same time period. None of the healthy male controls were first-degree relatives of any dcSSc subjects nor had any known autoimmune disease, based on a completed health status form prior to enrollment. The cohort of post-menopausal females with dcSSc was previously described [11]. The study was approved by the institutional review board (IRB) of the University of Pittsburgh.

### E2 measurement

Serum samples were collected at the time of the first visit and stored at − 80 °C. Serum samples were thawed on ice prior to E2 measurement. Each undiluted serum sample was measured in duplicate using an ELISA (Calbiotech ES180S, Spring Valley, CA) that has been previously validated against mass spectrometry [11]. The protocol for E2 measurement was followed as outlined by the manufacturer without deviation. Each duplicate serum E2 measurement was reported as average and used in the statistical analysis. We similarly measured serum E2 levels in healthy controls.

### Clinical information

Clinical information, including modified Rodnan skin score (mRSS), internal organ involvement, and time to death, was analyzed retrospectively in the dcSSc male cohort. The mRSS, which is validated to measure skin thickness, ranges from 0 to 3 for assessing uninvolved skin to severe skin thickness, respectively, in 17 assigned cutaneous surface areas [13]. The mRSS was performed by trained Scleroderma Center rheumatologists at the first visit. Skin thickness progression rate was calculated as previously described [14]. Determination of internal organ involvement was defined as previously described and included the heart (pericarditis, congestive heart failure (CHF), arrhythmias requiring prescription medication(s), and/or myocarditis), lungs (pulmonary arterial hypertension (PAH) or interstitial lung disease (ILD)), and kidney (scleroderma renal crisis (SRC)) [14]. Internal organ involvement occurring during longitudinal follow-up was also recorded, if known. Patients who were taking hormone supplements and/or had end-stage renal disease were excluded from the study due to the potential influences on serum E2 levels. All data were entered into the University of Pittsburgh Scleroderma Database. Time to death was calculated from the first Scleroderma Center visit.

### Autoantibody identification

At the time of enrollment, the SSc patients’ autoantibody profiles were determined from serum samples collected and analyzed by the Scleroderma Center Research laboratory [14, 15]. We tested for antibodies targeting RNA polymerase III (POL3), Scl-70, U1RNP, U11/U12 RNP, and centromere.

### Statistical analysis

Descriptive statistics were calculated for male dcSSc patients. Comparisons of serum E2 levels between dcSSc male patients with healthy controls and with dcSSc female patients were conducted using Wilcoxon rank sum tests. Additionally, evaluation of univariate associations between patient characteristics in dcSSc male patients was conducted using Pearson’s or Spearman’s rank correlation for continuous characteristics and the Wilcoxon rank sum test for categorical variables. The association between the occurrence of organ involvement including the lung, heart, and kidney involvement with serum E2 level was evaluated using a logistic regression approach. Univariate associations between skin thickness progression rate with serum E2 and other patient characteristics were examined using a linear regression approach. Univariate associations between time to death with serum E2 level and other variables of interest were examined using a Cox regression approach. Multivariable linear and Cox regression models were also developed for skin thickness progression rate and for time to death respectively, considering all variables that had univariate associations of *p* < 0.20 and two-way interactions between serum E2 level and other characteristics. The final models for these outcomes were selected using backward selection retaining all covariates with significance in the models of *p* < 0.10. The proportional hazards assumption in the Cox regression model was evaluated using the Grambsch-Therneau test [16]. For all analyses, statistical assumptions, including normality, were assessed, and if assumptions were not met, nonparametric approaches or appropriate transformations were employed. All analyses were conducted in SAS v. 9.4, with significance set as *p* < 0.05.

## Results

### Demographics

The study population included 83 male dcSSc patients who met enrollment criteria and 37 healthy controls. The mean age at which study patients’ serum E2 level was measured was 60.7 ± 7.9 years, and the mean E2 level was 30.6 ± 17.4 pg/mL. The mean disease duration at the time of serum E2 measurement was 1.19 ± 0.7 years. Given the limited number of patients positive for anti-centromere, anti-U1RNP, and anti-U11/U12 RNP, these were treated as “other autoantibody.” Patient characteristics for the dcSSc male cohort are summarized in Table 1. The demographics for the female dcSSc patient cohort were previously described [11].

**Table 1:** Characteristics of male patients with dcSSc. Continuous variables are reported as mean (SD) and categorical variables as *n* (%)

Patient characteristics	*n* = 83
Race (Caucasian)	77 (93)
Age at first symptom (years)	59.5 (8.0)
Disease duration from first symptom (years)	1.19 (0.7)
Baseline skin score	29.1 (11.3)
Serum E2 level (pg/mL)	30.6 (17.4)
Age of E2 measurement (years)	60.7 (7.9)
POL3 antibody (yes)	43 (51.8)
Scl-70 antibody (yes)	14 (18.1)
Other autoantibody (yes)	8 (6.0)

### Differences in serum E2 levels in dcSSc patients and healthy controls

**Fig. 1:**
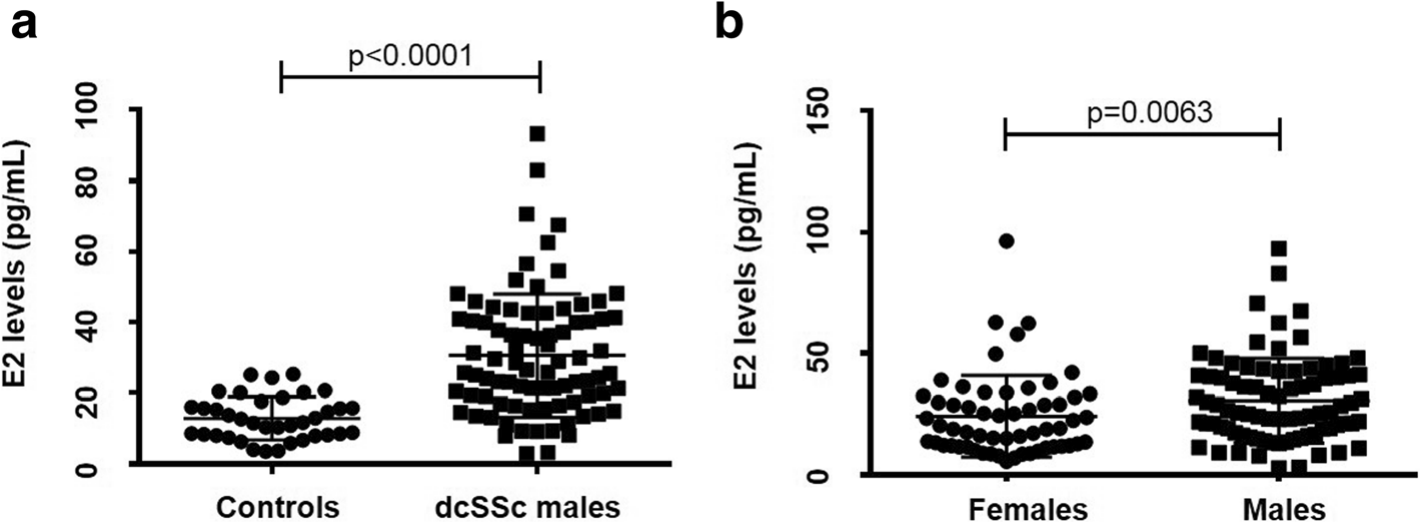
**a** Serum E2 levels in male healthy controls vs. male dcSSc patients. The difference between the groups was significant (*p* < 0.0001). **b** Serum E2 levels in male dcSSc patients vs. post-menopausal female dcSSc patients. The difference between the groups was significant (*p* = 0.0063). Line represents mean ± SD

Male patients with dcSSc had significantly higher serum E2 levels compared to healthy controls, with a mean of 30.6 pg/mL compared to 12.9 pg/mL and standard deviation (SD) of 17.4 pg/mL compared to 6.1 pg/mL, respectively (*p* < 0.0001) (Fig. 1a). Among male dcSSc patients, serum E2 was also compared by patient characteristics (Table 2). POL3-positive male patients had significantly lower serum E2 levels compared to those negative for that antibody (25.9 pg/mL ± 13.2 pg/mL vs. 35.7 pg/mL ± 19.9 pg/mL, respectively; *p* = 0.027).

**Table 2:** Mean serum E2 levels by patient characteristics. For categorical variables, E2 levels are reported as mean (SD) by group. For continuous variables, we report the Pearson correlation

Characteristic	Group	*n*	E2 (SD)	*p*
Age at measurement	Years	83	− 0.086	0.439
Disease duration	Years	83	− 0.116	0.297
Baseline skin score		83	0.054	0.573
POL3	No	40	35.7 (19.9)	0.027
	Yes	43	25.9 (13.2)	
Scl-70	No	69	29.3 (16.9)	0.103
	Yes	14	37.1 (18.9)	
Other autoantibody	No	75	31.4 (17.8)	0.207
	Yes	8	23.1 (11.9)	
Organ involvement				
Lung	No	38	32.0 (20.6)	0.488
	Yes	45	29.4 (14.3)	
Heart	No	76	29.4 (16.7)	0.037
	Yes	7	43.7 (20.4)	
Kidney	No	65	31.0 (17.7)	0.704
	Yes	18	29.2 (16.8)	

### Comparison of serum E2 levels in dcSSc male and post-menopausal female patients

Serum E2 levels of dcSSc male patients > 50 years old were also compared to serum E2 levels in the previously published cohort of post-menopausal female dcSSc patients [11]. Surprisingly, male dcSSc patients > 50 years old had significantly higher mean serum E2 levels compared to the mean serum E2 level of the post-menopausal female dcSSc patients previously reported (mean of 30.6 pg/mL ± 17.4 pg/mL vs. 24.2 pg/mL ± 16.7 pg/mL, respectively; *p* = 0.0063, Fig. 1b) [11].

### Association of serum E2 levels and internal organ involvement

The mean serum E2 level by internal organ involvement status in male dcSSc patients is shown in Table 2. Male patients with dcSSc and heart involvement had significantly higher serum E2 levels compared to those without heart involvement (43.7 pg/mL ± 20.4 pg/mL vs. 29.4 pg/mL ± 16.7 pg/mL, respectively; *p* = 0.037). No significant associations between serum E2 and lung or kidney involvement were noted.

### Association of serum E2 levels and skin thickness progression rate

In univariate analyses, skin thickness progression rate was positively correlated with age at the first symptom and age at which E2 was measured and was negatively correlated with disease duration (*p* = 0.006, 0.036, and < 0.001 respectively). Skin thickness progression rate was also associated with POL3 status with POL3-positive patients having significantly greater rates of progression relative to POL3-negative patients (*p* = 0.029) as previously reported [14, 17].

**Fig. 2:**
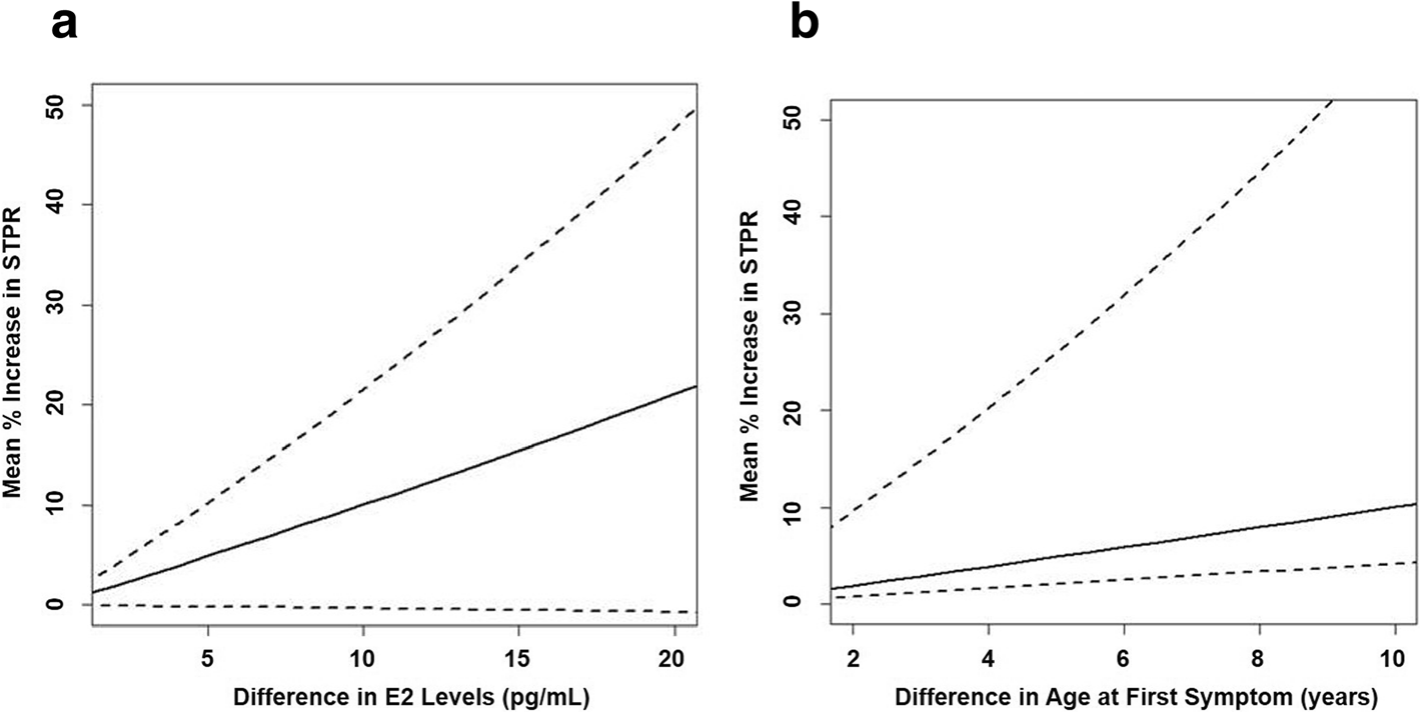
The estimated change in skin thickness progression rate (STPR) for the difference in serum E2 levels. The solid line is the percent increase in the STPR for the difference in serum E2 levels between patients (**a**) or difference in age at first symptom (**b**). The dashed lines are the 95% confidence interval boundaries for the percent increase in the STPR

In the multivariable model (Table 3), the skin thickness progression rate was natural log transformed to meet model assumptions. Age at first symptom, POL3, and E2 levels were included in the model and were all significant at *p* < 0.10. A 10-pg/mL increase in E2 levels was associated with a 10% increase in the skin thickness progression rate controlling for POL3 status and age at first symptom (95% confidence interval (CI) − 0.4–21.5% higher rate for 10-pg/mL increase in E2; *p* = 0.062, Fig. 2a). Patients who were positive for POL3 had a 42% higher skin thickness progression rate relative to those who were POL3 negative after controlling for other factors (95% CI 0.00–102% higher rate; *p* = 0.051).

**Table 3:** Multivariable models of the natural log of skin thickness progression rate. Skin thickness progression rate was natural log transformed to meet statistical assumptions

Variable	Percent difference (95% CI)	*p*
E2 level (10-pg/mL increase)	10.0 (− 0.40, 21.5)	0.062
POL3 (yes vs. no)	42.4 (0.00, 101.8)	0.051
Age at first symptom	13.4 (2.09, 25.9)	0.022

A 5-year increase in age at first symptom was associated with a 13% increase in the skin thickness progression rate controlling for POL3 status and E2 level (95% CI 2.1–25.9% higher rate for 10-pg/mL increase in E2; *p* = 0.022, Fig. 2b).

### Association of age of symptom onset, high vs. low serum E2 levels, and survival

**Fig. 3:**
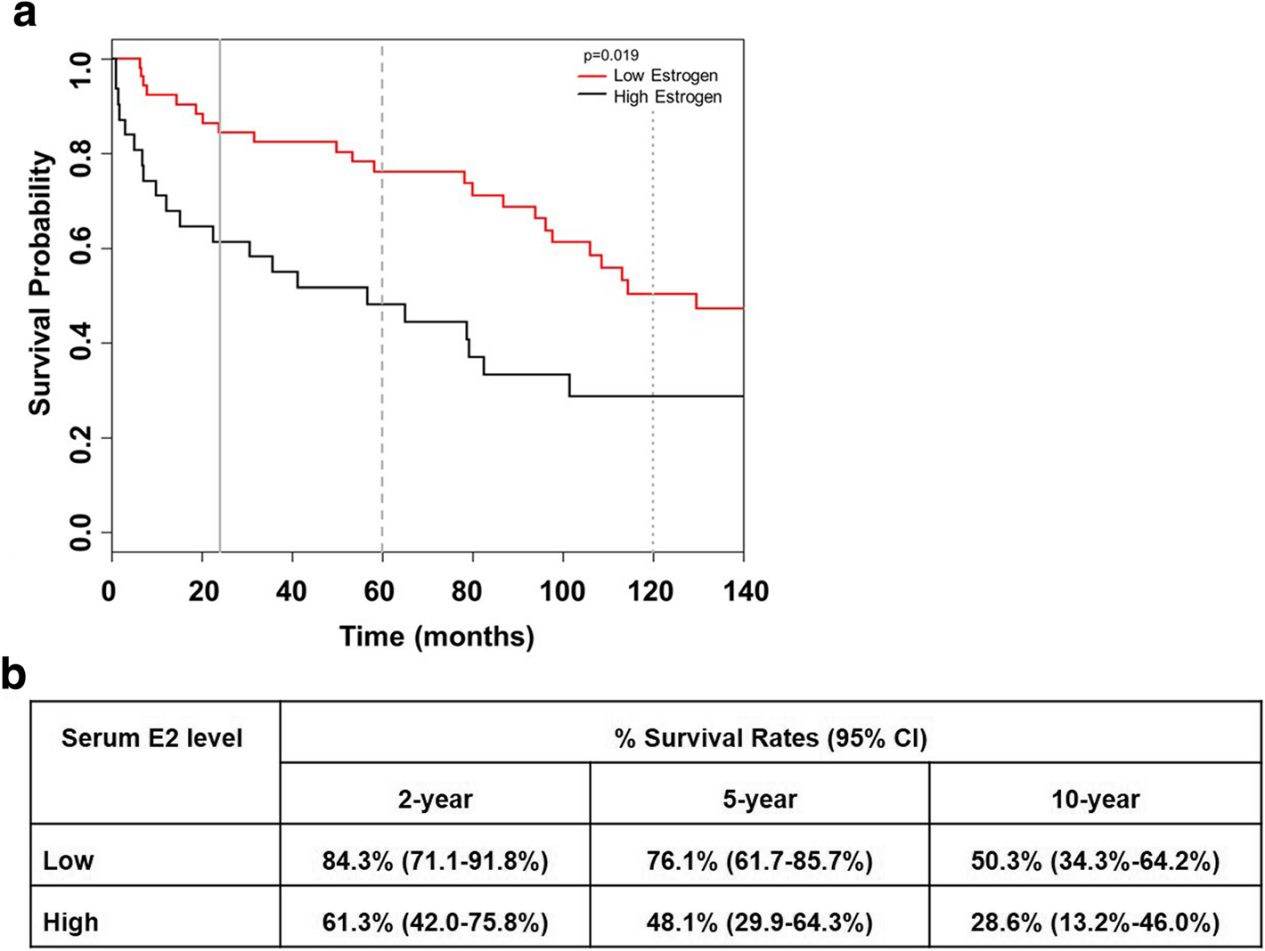
**a** Kaplan-Meier curve for the probability of survival by serum E2 group. The black line is the estimated survival probability for patients with high serum E2 levels, defined as E2 level greater than 36 pg/mL. The red line is the estimated survival probability for patients with low E2 levels. The vertical gray solid, long-dashed, and short-dashed lines are for 2-, 5-, and 10-year survival, respectively. The difference in survival between the low and high E2 groups was significant (*p* = 0.0190). **b** Corresponding 2-, 5-, and 10-year % survival rates with 95% CI

In univariate survival models, time to death in male dcSSc patients was significantly associated with the serum E2 level and age of symptom onset, with higher serum E2 levels and older age of onset being associated with an increased risk of death (*p* = 0.042 and 0.007, respectively; Table 4). Specifically, a 5-year increase in age of symptom onset was associated with a 28% increase in the risk of death (hazard ratio (HR) 1.28, 95% CI 1.07–1.52). A 10-pg/mL increase in serum E2 was associated with an 18% increase in the risk of death (HR 1.18, 95% CI 1.01–1.39, Table 4). The proportional hazards assumption for the univariate Cox regression model of time to death with serum E2 level was not met; therefore, we also considered serum E2 dichotomized into low and high E2 levels. The cut-point for low versus high serum E2 was selected as 36 pg/mL, based on the value that resulted in the smallest log-likelihood for a univariate Cox regression model. Patients with dcSSc with high serum E2 levels had an 89% increase in the hazard of death relative to dcSSc patients with low serum E2 levels, which was statistically significant (HR 1.89, 95% CI 1.11–3.22; *p* = 0.022). The Kaplan-Meier curves by the serum E2 group (low vs. high) and the estimated 2-, 5-, and 10-year percent survival rates divided by low and high serum E2 groups are shown in Fig. 3a and b, respectively. In univariate models, time to death was not significantly associated with race, baseline skin score, POL3 status, Scl-70 status, or organ involvement.

**Table 4:** HRs (95% CI) for univariate and multivariable Cox regression models of time to death

Variable	Models			
	Univariate		Multivariable	
	HR (95% CI)	*p*	HR (95% CI)	*p*
Age at first symptom (5-year increase)	1.28 (1.07–1.52)	0.007	1.36 (1.14–1.62)	< 0.001
E2 level (10-pg/mL increase)	1.18 (1.01–1.39)	0.042		0.555
Scl-70 status (yes)	1.77 (0.86–3.65)	0.124		0.006
Age at first symptom × Scl-70				< 0.001
Scl-70 yes vs. no (at 25th percentile E2 level (17.4))			0.16 (0.03–1.01)	
Scl-70 yes vs. no (at 50th percentile E2 level (25.8))			0.48 (0.12–1.89)	
Scl-70 yes vs. no (at 75th percentile E2 level (40.8))			3.47 (1.47–8.19)	
10-pg/mL increase in E2 level (for Scl-70 positive)			3.95 (2.08–7.49)	
10-pg/mL increase in E2 level (for Scl-70 negative)			1.06 (0.87–1.29)

**Fig. 4:**
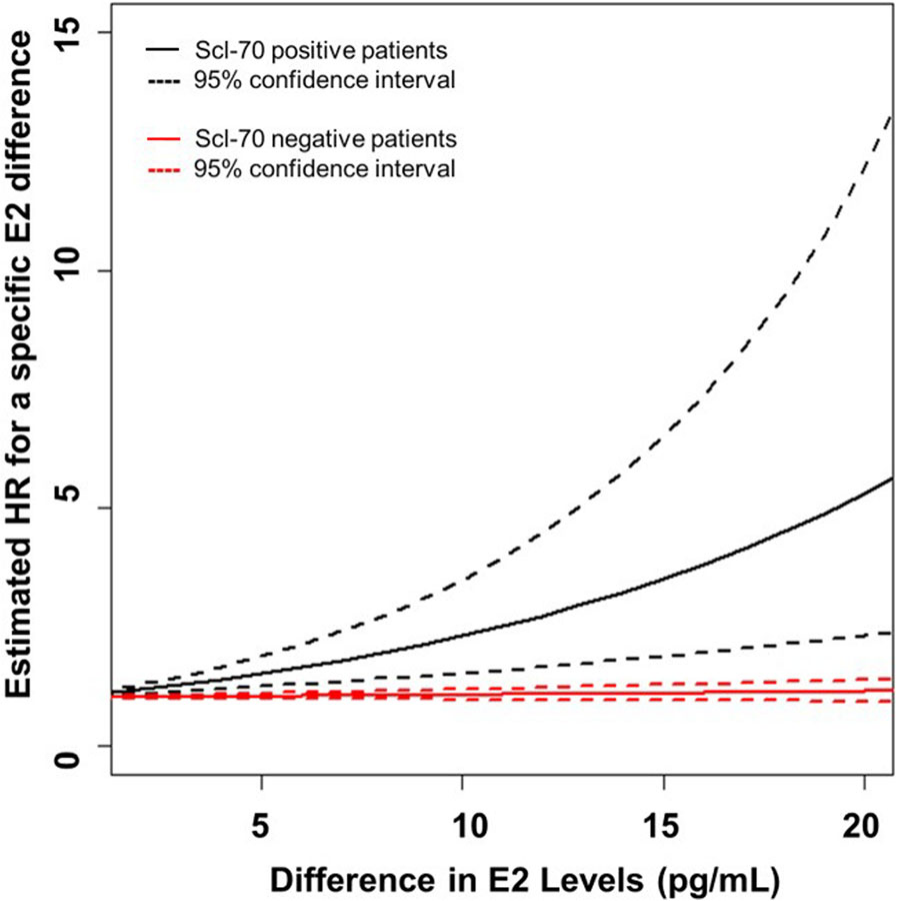
The estimated HR for the difference in serum E2 levels based on Scl-70 status. The solid black line is the estimated HR for the difference in serum E2 levels between Scl-70-positive patients. Dashed black lines are the 95% confidence interval boundaries for the HR. The solid red line is the estimated HR for the difference in serum E2 levels between Scl-70-negative patients. The dashed red lines are the 95% confidence interval boundaries for the HR

We also developed a multivariable Cox regression model as described in the “Methods” section. The final model included age at first symptom, serum E2 level, Scl-70 status, and the interaction between serum E2 levels and Scl-70 status. No other significant interactions were noted. All model assumptions, including proportional hazards, were checked and were found to be met. Similar to the univariate Cox model, older age of symptom onset was significantly associated with a 36% increased risk of death in male dcSSc patients after controlling for E2 serum levels and occurrence of Scl-70 (HR for a 5-year increase = 1.36; 95% CI 1.14–1.62; *p* < 0.001, Table 4). Due to the significant interaction between the occurrence of Scl-70 and serum E2 level, these covariates were interpreted together. Table 4 and Fig. 4 show the estimated HR for increasing differences in serum E2 level by Scl-70 status (black = Scl-70 positive, red = Scl-70 negative). The results from the model suggest that, given a patient is Scl-70 positive, increasing serum E2 level is associated with a greater risk of death, particularly as the difference in serum E2 levels increases. However, serum E2 has little to no impact on the risk of death in Scl-70-negative patients.

## Discussion

We and others previously demonstrated that E2 promotes ECM production in primary human dermal fibroblasts in vitro [11, 18], and our group demonstrated that E2 induces dermal thickening in an ex vivo human skin organ culture model [11]. The pro-fibrotic effect of E2 appears to be mediated through estrogen receptor α (ERα), since the use of ICI 182,780, an ERα antagonist, reduced E2-mediated skin thickening and ECM production [11].

The increased skin thickness measured by the mRSS in SSc patients is in part due to increased synthesis and deposition of ECM components (i.e., collagen and fibronectin) by fibroblasts [19]. Fibroblasts isolated from the skin and lung of SSc patients are activated to produce more ECM than corresponding fibroblasts from healthy individuals [19–21]. Studies show that dermal skin thickness and collagen content increase in women on hormone replacement therapy (HRT), which is typically estrogen based, compared to females not taking HRT [22–27]. Together, these findings suggest a role for E2 in dermal fibrosis and in the pathogenesis of SSc.

Our study demonstrates that male dcSSc patients have higher serum E2 levels compared to healthy males of similar age. Interestingly, dcSSc male patients also have higher serum E2 levels compared to dcSSc female patients of comparable age [11]. Previous studies examined differences in serum testosterone levels in SSc. A small study of 5 male SSc patients and 8 controls did not show a difference in serum testosterone levels in dcSSc or lcSSc male patients compared to healthy controls [28]. It is unclear whether free or total serum testosterone was measured in this study. However, a slightly larger study demonstrated significantly higher total serum testosterone and E2 levels in 10 male dcSSc patients compared to age-matched controls [29]. Thus, increased testosterone levels in dcSSc males, as in healthy males, likely drive the elevated serum E2 levels and may explain the difference we observed between dcSSc male and female patients. This is due to the fact that testosterone is enzymatically converted to estrogen via aromatase, suggesting increased aromatase levels and/or activity in dcSSc and providing an explanation for the increased circulating E2 levels in males with dcSSc.

Additionally, we found that serum E2 levels in post-menopausal lcSSc patients are significantly lower than those in post-menopausal dcSSc patients and comparable to levels detected in healthy controls (data not shown). This is consistent with the association of serum E2 levels and dermal fibrosis in dcSSc patients. Even though serum E2 levels were not tested in male lcSSc patients in this cohort, we hypothesize from our unpublished findings in lcSSc female patients that male lcSSc patients will likely have lower serum E2 levels compared to dcSSc male patients, paralleling the extent of skin involvement.

Increases in male dcSSc age and serum E2 levels correlated with an increase in the skin thickness progression rate. POL3 also positively correlated with skin thickness progression rate which supports previous studies that RNA POL3-positive patients have a higher mRSS and more rapid skin thickness progression rate [14, 17]. Additionally, there is a trend towards a positive association between serum E2 levels and the skin thickness progression rate, though it did not achieve statistical significance in this study, likely due to limited power. As previous data demonstrated that E2 increases ECM components in vitro and ex vivo [11], our data provides further support to the hypothesis that the rate of progression of dermal fibrosis may be affected by serum E2 levels in male dcSSc patients. However, this association needs to be confirmed in a larger cohort of dcSSc patients. Increasing age at first symptom also has a higher mean percent skin thickness progression rate. This is likely due to the fact that with increasing age, the higher serum E2 levels due to increased aromatization [10] promote increased dermal fibrosis and skin thickness progression rate.

Our data also show that male POL3-positive dcSSc patients have a significantly lower serum E2 level compared to male POL3-negative dcSSc patients. However, male POL3-negative dcSSc patients still have higher serum E2 levels compared to healthy controls. While POL3-positive dcSSc patients experience a higher skin score that peaks early [17], preliminarily, we found a more rapid decline in the skin score over time in male POL3-positive dcSSc patients in our cohort (data not shown). This suggests that a lower serum E2 level in male POL3-positive dcSSc patients may be a precursor to the skin thickness regression experienced in this subset of dcSSc patients.

Approximately half of the male dcSSc patients in our cohort were POL3 positive. This is comparable to a previous study from the University of Pittsburgh Scleroderma Center in which POL3 autoantibodies were detected in 45% of patients with dcSSc [30] and reflects the prevalence of POL3 positivity in the USA. Further, Caucasian males with SSc are more frequently POL3 positive compared to females or other ethnic groups [31].

We found increased serum E2 levels were associated with decreased survival in our cohort and this association was found to be exaggerated by Scl-70 antibody status with Scl-70-positive patients showing increased risk of death with increasing E2 levels. Although the patients’ cause of death was not evaluated in this study, older age and dcSSc are uniformly accepted risk factors for death in SSc cohorts [32]. Comorbid conditions also contribute to reduced survival in elderly patients. Heart involvement due to SSc is well recognized to be associated with poor survival [33–35]. Cardiac SSc is also associated with increased mortality in childhood-onset disease [36], and the presence of Scl-70 is associated with decreased survival in adults [37]. Both cardiac involvement and Scl-70 antibody were present in our adult cohort. We found cardiac involvement was also associated with increased serum E2 levels, which may have contributed to the decreased survival. The mean percent increase in skin thickness progression rate was higher in those with increasing serum E2 levels as well. Rapid skin thickness progression rate (> 45) is an independent risk factor for increased mortality and developed severe cardiac disease [14]. Therefore, it is possible that increased aromatization of testosterone to estrogen in older patients [10] could contribute to disease severity and increased risk of death.

Interestingly, a recent study found that male sex is a predictor in the development of pulmonary hypertension and heart dysfunction in the EUSTAR cohort [38]. Additionally, inhibition of estrogen signaling through its classical receptors (ER alpha and beta) prevented PAH in mice, while both fulvestrant and anastrozole prevented and ameliorated PAH in mice [39]. While our study found an association between heart involvement and serum E2 levels in male dcSSc patients in the current cohort, lung involvement, which included PAH and/or ILD, was not associated with serum E2 levels. Future studies are required to assess an association with PAH and serum E2 levels and to determine, prospectively, if E2 level is a predictor for PAH.

Serum and plasma levels of estrogens have been examined in rheumatoid arthritis (RA) and systemic lupus erythematosus (SLE). In RA, one study showed that serum levels of estrone sulfate (the precursor of estrone and E2) did not differ between patients and healthy controls [40]. Yet, lower androgen concentrations and higher concentrations of hydroxylated estrogens were detected locally in the synovial fluid of RA patients, indicating aromatization is occurring in the inflamed joint [41]. In SLE patients, higher plasma levels of estrogens were detected compared to androgens, with plasma estrogens directly correlating with tissue aromatase activity [42]. These estrogen-androgen imbalances in RA and SLE have been implicated in disease pathogenesis, but there are few correlative/associative studies with estrogen and clinical variables in either of these diseases [43, 44].

We recognize there are limitations to our study. Our cohort size is somewhat small for rheumatic disease studies, but our findings are impactful since the number of male dcSSc patients older than 50 years of age is large due to the fact that the Pittsburgh Scleroderma Serum Bank and Database dates back to the 1970s. Longitudinal follow-up to detect late development of internal organ involvement could be extended beyond the writing of this manuscript. Further, as with all studies, validation of our findings in additional SSc patient cohorts would further strengthen our conclusions.

In summary, serum E2 levels are increased in dcSSc. These data, in conjunction with the reported pro-fibrotic effects of E2 in the skin [11], suggest that E2 may be a viable therapeutic target in dcSSc. Several E2/estrogen receptor antagonists and aromatase inhibitors are currently in use for the treatment of breast cancer and could readily be repurposed for the treatment of patients with dcSSc.

## Conclusions

Male dcSSc patients > 50 years old have significantly elevated serum E2 levels compared to healthy controls and post-menopausal females with dcSSc. Higher serum E2 levels are associated with cardiac involvement, decreased survival, and increased risk of death in Scl-70-positive patients. These initial findings provide insights into the potential impact of elevated E2 levels in dcSSc and may explain, at least in part, the increased mortality in older male patients with dcSSc.
